# Treatment with a JAK1/2 inhibitor ameliorates murine autoimmune cholangitis induced by IFN overexpression

**DOI:** 10.1038/s41423-022-00904-y

**Published:** 2022-08-30

**Authors:** Tihong Shao, Patrick S. C. Leung, Weici Zhang, Koichi Tsuneyama, William M. Ridgway, Howard A. Young, Zongwen Shuai, Aftab A. Ansari, M. Eric Gershwin

**Affiliations:** 1 Department of Rheumatology and Immunology, The First Affiliated Hospital of Anhui Medical University, Hefei, China; 2 Division of Rheumatology, Allergy, and Clinical Immunology, School of Medicine, University of California, Davis, CA USA; 3 Department of Pathology and Laboratory Medicine, Institute of Biomedical Sciences, Tokushima University Graduate School, Tokushima, Japan; 4 Center for Cancer Research, National Cancer Institute—Frederick, Frederick, MD USA

**Keywords:** Primary biliary cholangitis, Autoimmunity, Interferons, Janus Kinase Inhibitors, Ruxolitinib, Autoimmunity, Immunotherapy

## Abstract

The interferon (IFN) signaling pathways are major immunological checkpoints with clinical significance in the pathogenesis of autoimmunity. We have generated a unique murine model named ARE-Del, with chronic overexpression of IFNγ, by altering IFNγ metabolism. Importantly, these mice develop an immunologic and clinical profile similar to patients with primary biliary cholangitis, including high titers of autoantibodies and portal inflammation. We hypothesized that the downregulation of IFN signaling pathways with a JAK1/2 inhibitor would inhibit the development and progression of cholangitis. To study this hypothesis, ARE-Del^+/−^ mice were treated with the JAK1/2 inhibitor ruxolitinib and serially studied. JAK inhibition resulted in a significant reduction in portal inflammation and bile duct damage, associated with a significant reduction in splenic and hepatic CD4^+^ T cells and CD8^+^ T cells. Functionally, ruxolitinib inhibited the secretion of the proinflammatory cytokines IFNγ and TNF from splenic CD4^+^ T cells. Additionally, ruxolitinib treatment also decreased the frequencies of germinal center B (GC B) cells and T follicular helper (Tfh) cells and led to lower serological AMA levels. Of note, liver and peritoneal macrophages were sharply decreased and polarized from M1 to M2 with a higher level of IRF4 expression after ruxolitinib treatment. Mechanistically, ruxolitinib inhibited the secretion of IL-6, TNF and MCP1 and the expression of STAT1 but promoted the expression of STAT6 in macrophages in vitro, indicating that M1 macrophage polarization to M2 occurred through activation of the STAT6-IRF4 pathway. Our data highlight the significance, both immunologically and clinically, of the JAK/STAT signaling pathway in autoimmune cholangitis.

## Introduction

Type I and II interferons (IFNs) have diverse effects on both innate and adaptive immune cells. The results from a plethora of studies have demonstrated that IFNs are involved in the pathogenesis of many autoimmune diseases. Type I IFNs participate in antiviral responses mediated by a wide range of cell types, can increase major histocompatibility complex expression and antigen presentation [1] and can also recruit immune cells to specific tissues in response to the secretion of chemokines and cytokines within the local microenvironment. Type I IFNs can activate natural killer (NK) cells and antigen-presenting dendritic cells (DCs), B cells, and CD4^+^ and CD8^+^ T cells [2–7] and affect monocyte and/or macrophage function and differentiation [8]. Type II IFN (IFNγ) has weaker antiviral effects than type I IFNs but acts on a majority of similar cell lineages and induces similar potent effects, such as increasing major histocompatibility complex expression, antigen presentation and chemokine production. IFNγ augments innate immune responses, including inducing monocytes into DCs and macrophages and boosting the function of type 1 innate lymphoid cells [9, 10]. IFNγ is also involved in the generation of T follicular helper (Tfh) cells, the formation of germinal centers (GCs) and the induction of pathogenic autoantibodies, contributing to autoimmune diseases [11, 12].

IFNs perform their effector functions through the activation of the JAK-STAT pathway [13, 14]. The JAK/STAT pathway constitutes a rapid membrane-to-nucleus signaling cascade, including extracellular cytokines, intracellular JAK that transmit signals, and transcription factors. More than 50 types of cytokines can bind membrane receptors; four are JAK proteins (JAK1, JAK2, JAK3, and TYK2), and seven are STAT proteins (STAT1, STAT2, STAT3, STAT4, STAT5a, STAT5b, and STAT6). Individual cytokines bind to the receptor complex, which can associate with more than one JAK and subsequently activate one or more STAT proteins. Binding of cytokines to receptors primes the signaling process through a cascade of reactions: JAK phosphorylation is followed by STAT phosphorylation to form STAT homodimers or heterodimers, which enter the nucleus to initiate the expression of target genes. The specific effector function depends on the corresponding cytokine. The JAK/STAT pathway is important in many aspects of the immune system [15, 16]. Several IFN risk allele variants are involved in the induction of prolonged IFN signaling in a variety of autoimmune diseases, such as systemic lupus erythematosus (SLE), rheumatoid arthritis (RA), systemic sclerosis, vasculitis and primary biliary cholangitis (PBC) [17–24]. Experimental evidence has documented the role of IFNγ in the pathogenesis of PBC [24, 25]. To further examine the contribution of IFNs in PBC, we generated a mouse model with chronic overexpression of IFNγ by replacing the AU-rich element (ARE) in the 3′ UTR of IFNγ mRNA with random nucleotides. These mice exhibit increased and chronic expression of IFNγ in both homozygous and heterozygous ARE replacement mice. ARE-Del mice display distinct clinical and pathological features of autoimmune cholangitis that are characteristic of human PBC, including female predominance, biliary lymphocyte infiltration, destruction of small biliary ducts, increased serum bile acids and the presence of anti-mitochondrial antibodies (AMA). The observation that female ARE-Del mice display type I and II IFN signaling abnormalities as a component of female predominant autoimmune cholangitis further underscores the role of type I IFN signaling and its interaction with type II IFN as an essential factor for the pathology [26]. Noting that JAK1 and JAK2 are downstream signaling checkpoints of IFNs, our observation that JAK1, JAK2, TyK2 and STAT1 are expressed at higher levels in liver tissue of ARE-Del mice than in control mice [26] suggests that type I and type II IFNs, through their cognate receptors, can activate the JAK/STAT signaling pathways and regulate immune cell differentiation and activation [16, 27, 28]. Taken together, our previous findings strongly support the role of IFNs in the pathogenesis of PBC.

Inhibitors of the JAK family have demonstrated clinical benefits in the treatment of RA and other inflammatory disorders [29–31]. Upadacitinib, a selective JAK1 inhibitor, proved efficacious in patients with active ankylosing spondylitis [32]. Upadacitinib monotherapy or combination with conventional synthetic disease-modifying antirheumatic drugs (csDMARDs) significantly improved the clinical and functional outcomes in patients with RA who had an inadequate response to DMARDs [33]. Baricitinib, an oral selective JAK1 and JAK2 inhibitor, was efficacious and well tolerated in patients with active SLE who were refractory to standard therapy [34]. A recent genome-wide meta-analysis identified the JAK/STAT signaling pathway as a potential therapeutic target for PBC [35]. A phase II, proof-of-concept, double-blind, randomized, parallel placebo-controlled study reported that baricitinib, a JAK1/2 inhibitor, administered to PBC patients can decrease ALP and markers of inflammation and improve pruritus and self-reported depression compared with placebo. Although only 2 patients were enrolled in this study and the treated subject’s ALP rebounded to pretreatment levels within 4 weeks of treatment discontinuation, the initial encouraging response to baricitinib suggests a prominent mechanistic role of JAK inhibitors in the treatment of PBC [36]. Herein, we hypothesize that downregulating the JAK/STAT signaling pathway in immune cells may provide a viable strategy for modulating IFN-mediated liver autoimmunity in PBC. Since ruxolitinib is an FDA-approved JAK1/2 inhibitor for the treatment of RA and myelofibrotic diseases [37], we carried out the studies reported herein to determine its therapeutic effect on the disease process in ARE-Del mice.

## Materials and methods

### Protocol for the use of ARE-Del^+/−^ mice

**Fig. 1:**
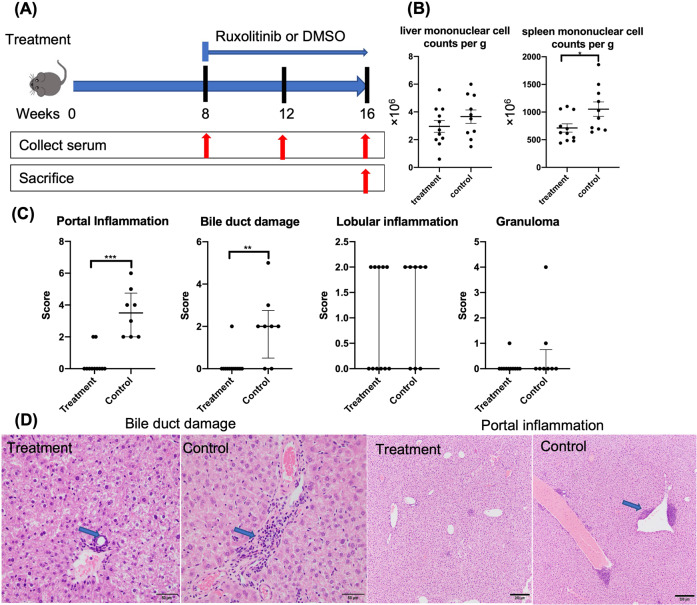
Ruxolitinib treatment improved liver pathology in ARE-Del^+/−^ mice. **A** The treatment protocol utilized in the treatment of ARE-Del^+/−^ mice. Groups of mice were injected intraperitoneally with either ruxolitinib or the control carrier DMSO. **B** Representative liver and spleen mononuclear cell counts in ruxolitinib-treated (*n* = 11) and control group mice (*n* = 10). **C** Graphical summary of the histological scores of disease severity between the treatment (*n* = 11) and control groups (*n* = 8). **D** Representative displays of H&E-assisted liver pathology from the treatment and control groups (original magnification ×40). *p < 0.05, **p < 0.01, ***p < 0.001. **→** Lymphocyte infiltration in the portal and biliary ducts is significantly reduced in treated mice compared with control mice

ARE-Del^+/−^ mice were generated as described previously [18]. Experiments were conducted under the approval of the University of California Animal Care and Use Committee. Female ARE-Del^+/−^ mice at 8 weeks of age were matched for age and sex and randomly assigned to either the treatment or control group. Mice assigned to the treatment group were administered 100 mg/kg/day ruxolitinib (MedChemExpress, Monmouth Junction, NJ) in 1.5% DMSO by intraperitoneal injection. At 8 weeks after the first treatment, animals were sacrificed, and sera and tissue samples (liver and spleen) were collected for serological analysis of AMA, gene expression, cellular immunological analysis and liver pathology analysis. Animals treated with DMSO only were studied in parallel as controls (Fig. 1A). All experiments were performed in group sizes of 8–11, and the numbers in each experiment are noted in the figure legends.

### Histopathology

Tissue isolation and slide preparation were performed as previously described [38, 39]. Hematoxylin and eosin staining and histological scoring were performed as described previously [26]. The liver histological score criteria were primarily based on the level of severity and frequency of pathological changes relative to a score from a control specimen in a blinded test [26]. The specific scoring scale is shown in Table S1.

### Cell isolation for flow cytometry analysis

Immediately after sacrifice, mononuclear cells were harvested from the liver and spleen as previously described [40, 41]. After washing the cells with ice-cold PBS-0.2% bovine serum albumin (BSA), the cells were incubated with Fc-block (eBioscience, San Diego, CA) for 15 min in PBS-0.2% BSA at 4 °C, washed again and stained with the appropriate cocktail of monoclonal antibodies for 30 min at 4 °C. For intracellular staining, cells were fixed with Foxp3 Fixation/Permeabilization working solution for 30 min at 4 °C, washed with BD Perm/Wash buffer, and then incubated with antibody cocktails diluted in BD Perm/Wash buffer. Monoclonal antibodies for staining of cell-surface markers included antibodies specific for TCR**β** (H57-597), CD4 (GK1.5), CD8**α** (53-6.7), C-X-C chemokine receptor type CXCR5 (2G8), programmed cell death 1 (PD-1; 29 F.1A12), B220 (RA3-6B2), CD95 (FAS, Jo2), GL-7 (GL7), NK1.1 (PK136), F4/80 (BM8), CD80 (16-10A1), CD206 (C068C2) and IRF4 (E8H3S). Monoclonal antibodies for the staining of intracellular markers included Foxp3 (FJK-16s), IFN**γ** (XMG1.2), and IL-17A (TC11-18H10.1). All reagents were purchased from BioLegend (San Diego, CA) except for antibodies specific for F4/80 (BM8) and IRF4 (E8H3S), which were purchased from eBioscience (San Diego, CA) and Cell Signaling (Danvers, MA), respectively. Predetermined optimal dilutions were used throughout with recommended positive and negative controls. Data were acquired on an LSRII flow cytometer (BD Biosciences, San Jose, CA). Data were analyzed using FlowJo software (Treeland, Ashland OR). All antibodies used are listed in Supplementary Table S2.

### In vitro cultures of CD4^+^ T cells and macrophages

Spleen cells were collected from 20-week-old female ARE-Del^+/−^ mice. Mononuclear cells were isolated, and CD4^+^ T cells were purified by positive selection with microbeads and MiniMacs separation columns. CD4^+^ T cells were incubated in 96-well plates (1 × 10^5^ cells/well) in the presence of magnetic beads (Dynabeads Mouse T-Activator CD3/CD28, Thermo Fisher Scientific, Waltham, MA) at a bead-to-cell ratio of 1:1 with varying concentrations (0, 0.1, 1, 5, and 10 µM) of ruxolitinib for 3 days. Macrophages were derived from bone marrow cells. Briefly, bone marrow cells were isolated from 20-week-old female wild-type (WT) mice and propagated for 7 days in macrophage complete medium (DMEM/F12-10 medium containing 10% fetal bovine serum (HyClone, Logan, UT) with 20 ng/ml recombinant GM-CSF (Bio-Techne Corporation, Minneapolis, MN). Adherent cells were harvested on day 7 and incubated in a 6-well plate (1 × 10^6^ cells/well) with varying concentrations (0, 1, 5, and 10 µM) of ruxolitinib for 3 days. Thereafter, the supernatant and the cells were collected and analyzed for specific cytokines, proteins and gene expression.

### RNA isolation and gene expression analysis

Total RNA was isolated using a Purelink RNA Mini Kit (Invitrogen, Waltham, MA) and reverse transcribed into cDNA using an iScript cDNA synthesis kit (BIO-RAD, Hercules, CA). Quantitative real-time PCR was performed with corresponding primers (Table S3, Invitrogen) specific for murine IFNγ, IL-6, TNF**α**, and TGFβ mRNA expression in tissues from the livers of ARE-Del^+/−^ mice. The data on the levels of gene expression were normalized to GAPDH.

### Determination of AMA

Serum AMA was detected using our standard enzyme-linked immunosorbent assay (ELISA) against recombinant proteins of the pyruvate dehydrogenase complex E2 subunit (PDC-E2) as described, with known positive and negative controls and standardized recombinant PDC-E2 [42, 43].

### Detection of cytokines

The levels of the cytokines IFNγ, IL-6, IL-17, TNF, IL-2, IL-4 and IL-10 were measured using the Cytometric Bead Array (BD Biosciences) [44] on an LSRII flow cytometer (BD Biosciences) and were analyzed using FlowJo software.

### Protein extraction and expression analysis

Macrophages (1 × 10^6^ cells) cocultured with various concentrations of ruxolitinib (0, 1, 5, and 10 µM) were lysed directly in 4 × LDS loading buffer. Equal amounts of cell lysates were resolved on Nu-PAGE gels; transferred to nitrocellulose membranes; probed with STAT1 antibody, phospho-Stat1 (Ser727) antibody, STAT6 antibody and β-actin (D6A8) rabbit mAb (HRP conjugate) (Cell Signaling); washed; and the reactivity was determined with the corresponding HRP-conjugated secondary antibodies and analyzed by ECL chemiluminescence (ProSignal® Pico ECL Reagent, Genesee Scientific, San Diego, CA). All antibodies used are listed in Supplementary Table S4.

### Statistical analysis

Datasets between the treatment and control groups were analyzed for statistical significance by the two-tailed Mann‒Whitney *U* test, unpaired Student’s *t* test or one-way ANOVA using GraphPad Prism software (GraphPad Software Inc., La Jolla, CA). Data are listed as n.s. (nonsignificant) or with asterisks as follows: *p < 0.05, **p < 0.01, ***p < 0.001, or ****p < 0.0001.

## Results

### Ruxolitinib treatment improved the overall liver pathology

After 8 weeks of treatment, a significant decrease in the cell count of mononuclear cells from the spleen, but not the liver, was detected in ruxolitinib-treated mice compared to controls (Fig. 1B). To evaluate the efficacy of ruxolitinib in ARE-Del^+/−^ mice, we first and foremost assessed liver pathology. Liver pathology was evaluated by analysis of the small bile duct architecture, biliary cell damage, portal inflammation, lobular inflammation, and frequencies of granulomas. Compared with that in control mice, liver inflammation was ameliorated in ruxolitinib-treated mice. While lymphocyte infiltration within the portal area and small bile duct regions was markedly reduced in ruxolitinib-treated mice, the degrees of lobular inflammation and granuloma formation were similar between the two groups (Fig. 1C, D). Taken together, the results demonstrated that treatment of ARE-Del^+/−^ mice with ruxolitinib led to significant reductions in biliary damage and portal inflammation.

### Ruxolitinib reduced the gene expression of proinflammatory cytokines (IFNγ and IL-6) in liver tissues

**Fig. 2:**
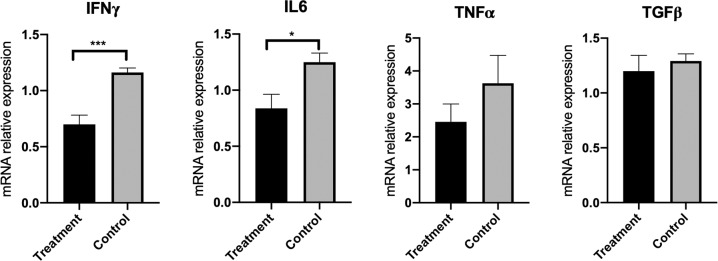
Ruxolitinib reduced the expression of proinflammatory cytokine genes in the livers of ARE-Del^+/−^ mice. RNA was isolated from the livers of ruxolitinib-treated mice (*n* = 11) and control mice (*n* = 8). The levels of mRNA expression of the proinflammatory genes (IFNγ, IL-6, and TNF**α**) and, for purposes of comparison, TGFβ were quantified by real-time PCR. The data displayed are expressed as the mean ± SEM. *p < 0.05, ***p < 0.001

To determine whether the change in liver pathology could be mediated by shifting the cytokine pattern, we measured the influence of ruxolitinib treatment on the levels of a selected number of proinflammatory and anti-inflammatory genes in liver tissues isolated from ARE-Del^+/−^ mice. The levels of the IFNγ and IL-6 gene expression were markedly decreased following treatment with ruxolitinib (Fig. 2). The level of TNF**α** expression was also lower in the ruxolitinib treatment group than in the control group. In addition, the relative expression of the anti-inflammatory cytokine TGFβ was comparable between the treatment and control groups.

### Ruxolitinib treatment decreased the frequencies of T cells and the production of proinflammatory cytokines but increased the frequencies of FOXP3+ Treg cells and the production of IL-2

**Fig. 3:**
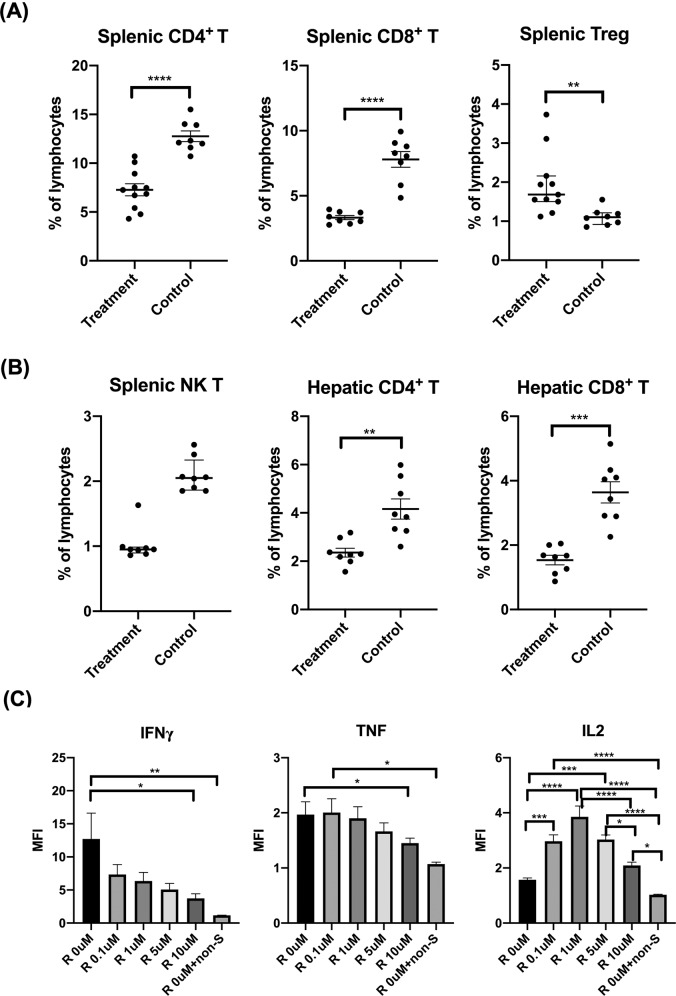
Ruxolitinib treatment decreased the frequencies of T cells and the production of proinflammatory cytokines but increased the frequencies of FOXP3+ Treg cells and the production of IL-2. **A** Flow cytometric analysis of splenic CD4+ T, CD8+ T and Treg cells from mice treated with ruxolitinib (*n* = 8–11) or controls (*n* = 8). **B** Flow cytometric analysis of splenic NK T cells and hepatic CD4+ T and CD8+ T cells from mice treated with ruxolitinib (*n* = 8) or controls (*n* = 8). **C** Representative results of IFNγ, TNF and IL-2 levels in splenic CD4+ T cells isolated from ARE^+/−^ (females/20 weeks) mice following in vitro treatment with ruxolitinib, as determined by CBA and flow cytometry. “R 0 µM + non-S” indicates ruxolitinib at 0 µM in the absence of anti-CD3/CD28. The data are representative of 3–5 independent experiments. One-way ANOVA, *p < 0.05, **p < 0.01, ***p < 0.001, ****p < 0.0001. MFI mean fluorescence intensity

Our previous study supports that T cells, especially CD4^+^ T cells, play a key role in the pathogenesis of autoimmune cholangitis in the ARE-Del model [26]. To examine the effect of ruxolitinib on T cells, we analyzed the subsets of T cells, including CD4^+^ T, CD8^+^ T, Treg and NK T cells, in the spleen and liver. Flow cytometry analysis revealed that treatment of ARE-Del^+/−^ mice with ruxolitinib decreased the frequencies of splenic CD4^+^ T, CD8^+^ T and NK T cells. Interestingly, a significant augmentation of splenic Treg cells was detected in ruxolitinib-treated mice compared with splenic cells from the control mice (Fig. 3A, B). In addition, ruxolitinib also decreased the frequencies of hepatic CD4^+^ T and CD8^+^ T cells compared with those in control mice (Fig. 3B).

To further examine the direct impact of ruxolitinib on T cells, splenic CD4^+^ T cells isolated from ARE-Del^+/−^ mice were cocultured with ruxolitinib (in the presence of CD3-CD28 beads) for 3 days. In vitro treatment of ARE-Del^+/−^ splenic CD4^+^ T cells with ruxolitinib led to the inhibition of IFNγ and TNF production, but increased production of IL-2 was detected in the culture supernatant when compared with untreated splenic CD4^+^ T cells from ARE-Del^+/−^ mice. Moreover, the decrease in IFNγ and TNF levels correlated with increasing concentrations of ruxolitinib (Fig. 3C). In contrast, the levels of IL-2 peaked at 1 µM ruxolitinib and then gradually declined with increasing concentrations of ruxolitinib. Altogether, the results indicated that ruxolitinib can inhibit the production of the proinflammatory cytokines IFNγ and TNF, which partly accounts for the improved liver pathology in ruxolitinib-treated ARE-Del^+/−^ mice.

### Ruxolitinib treatment decreased the frequencies of GC B cells and Tfh cells and led to lower serological AMA levels

**Fig. 4:**
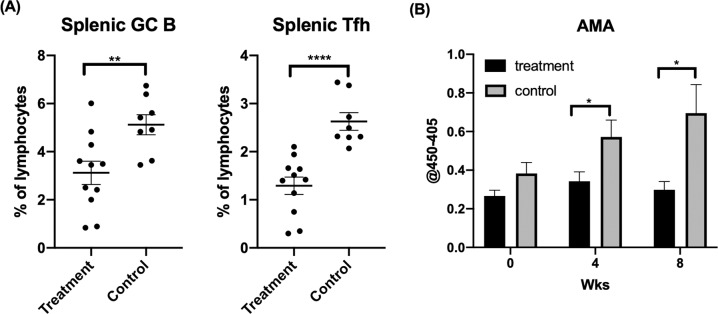
Ruxolitinib treatment decreased the frequencies of GC B cells and Tfh cells and led to lower serological AMA levels. **A** Flow cytometric analysis of splenic GC B and Tfh cells from mice treated with ruxolitinib (*n* = 11) or controls (*n* = 8) treated with carrier alone. **B** Levels of AMA before and at 4 and 8 weeks after ruxolitinib treatment in groups of ARE-Del^+/−^ mice treated with ruxolitinib (*n* = 11) or mice treated with the control carrier (*n* = 11). *p < 0.05, **p < 0.01, ****p < 0.0001

GC B and Tfh cells are critical in the induction of autoimmune cholangitis, as excessive GC formation is accompanied by high levels of AMA in ARE-Del^+/−^ mice [38]. To examine the effect of ruxolitinib on GC B cells, Tfh cells and AMA levels, we compared the frequencies of GC B and Tfh cells by flow cytometry and AMA by ELISA between ruxolitinib-treated and untreated control ARE-Del^+/−^ mice. The frequencies of GC B and Tfh cells in the spleen were dramatically reduced in ARE-Del^+/−^ mice compared with controls (Fig. 4A).

Moreover, we monitored the serum levels of AMA following the administration of ruxolitinib. The AMA level was markedly decreased at 4 and 8 weeks after the administration of ruxolitinib in ARE-Del^+/−^ mice but not in untreated controls (Fig. 4B). These findings indicate that ruxolitinib can ameliorate the accumulation of GC B cells and Tfh cells in spleens and can suppress AMA production in ARE-Del^+/−^ mice.

### Ruxolitinib treatment decreased the number of liver and peritoneal macrophages and was associated with a shift of M1 to M2 polarization through activation of IRF4

**Fig. 5:**
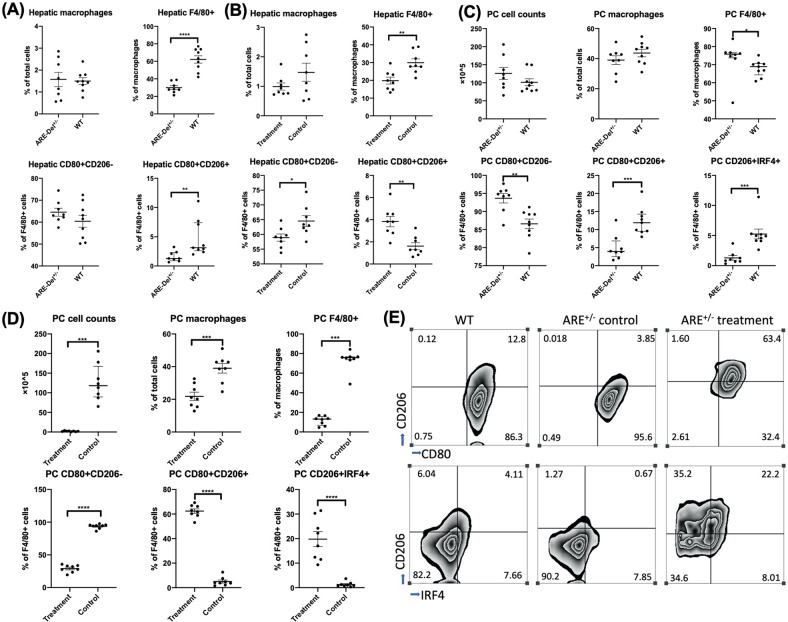
Ruxolitinib treatment decreased the number of liver and peritoneal cavity macrophages and was associated with a shift of M1 to M2 polarization through activation of IRF4. Flow cytometric analysis of hepatic macrophages, F4/80+ cells, M1 macrophages (CD80+CD206−), and M2 macrophages (CD80+CD206+) from ARE-Del^+/−^ mice (*n* = 8) and WT mice (*n* = 9) (**A**) or treatment (*n* = 8) and control mice (*n* = 8) (**B**). Peritoneal cavity (PC) cell counts and flow cytometric analysis of PC macrophages, F4/80+ cells, M1 macrophages, M2 macrophages, and CD206+IRF4+ macrophages from ARE-Del^+/−^ mice and WT mice (**C**) or treatment and control mice (**D**). **E** Representative flow image of M1 and M2 macrophages and CD206+IRF4+ cells from WT, ARE-Del^+/−^ treatment and control mice. All the gates are based on the F4/80+ population. One-way ANOVA, *p < 0.05, **p < 0.01, ***p < 0.001, ****p < 0.0001

Hepatic macrophages have been implicated in the pathogenesis of murine autoimmune cholangitis [45]. Macrophages were affected by the JAK1 selective kinase inhibitor and pan-JAK inhibitor tofacitinib in an experimental colitis model [46]. Here, we examine the effect of ruxolitinib on macrophage activation and signaling in vivo. Ruxolitinib treatment decreased the number of macrophages both in the liver and in the peritoneal cavity (PC) and was associated with an M1 to M2 polarization shift (Fig. 5B, D). Analysis of the subpopulations of macrophages in liver tissues and in the PC between the ARE-Del^+/−^ mice and age/sex-matched WT mice showed that while M1 macrophages were the predominant subpopulation in both the liver tissues and the PC of ARE-Del^+/−^ mice, there were more M2 macrophages in WT mice (Fig. 5A, C). The number and percentage of macrophages were notably decreased in the livers and PC of ARE-Del^+/−^ mice treated with ruxolitinib compared to control ARE-Del^+/−^ mice and WT mice (Fig. 5). Furthermore, F4/80+ macrophages expressed increased M2-associated markers, indicating a phenotypic shift from M1 to M2 macrophages both in liver tissues and the PC of ARE-Del^+/−^ mice treated with ruxolitinib when compared with controls and WT mice. This M1 to M2 shift in liver and PC macrophages reversed the M1:M2 ratio in ARE-Del^+/−^ mice (Fig. 5B, D). IRF4 is a key transcription factor that controls M2 macrophage polarization [47, 48]. To explore the specific signaling of M2 macrophage polarization in our study, we tested the expression of IRF4 in macrophages. The flow data results demonstrated that there was higher expression of IRF4 in WT mice than in ARE-Del^+/−^ mice (Fig. 5C). Notably, the expression level of IRF4 was markedly increased upon ruxolitinib treatment relative to that in ARE-Del^+/−^ control mice and WT mice (Fig. 5C, D), suggesting that M2 macrophage polarization relies on the activation of IRF4.

### Ruxolitinib treatment inhibited the production of proinflammatory cytokines and the expression of STAT1 but promoted the expression of STAT6 in macrophages in vitro

**Fig. 6:**
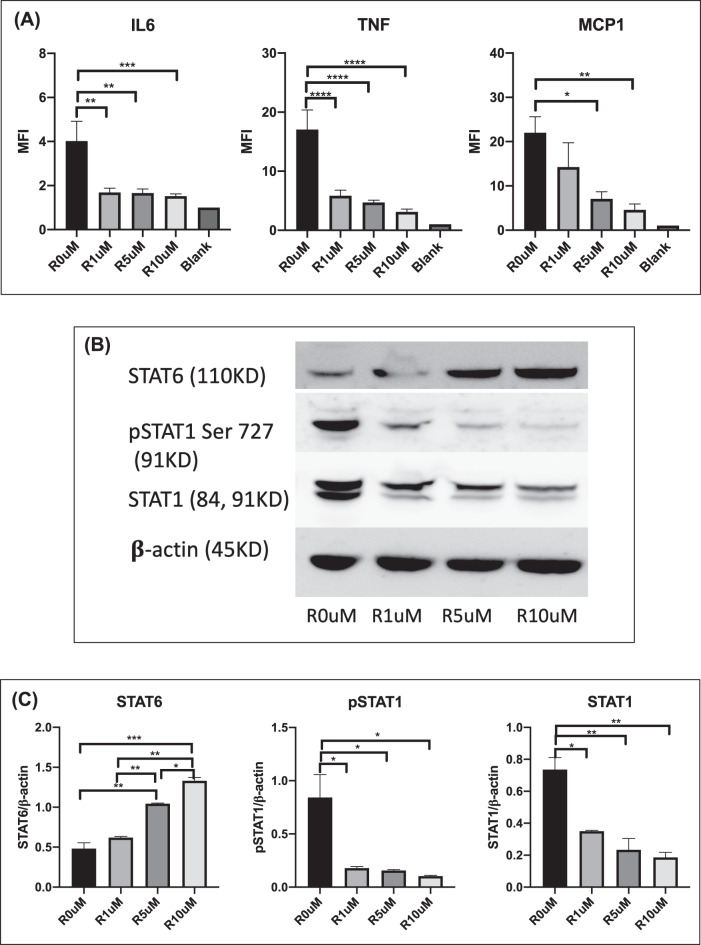
Ruxolitinib treatment inhibited the production of proinflammatory cytokines and the expression of STAT1 but promoted the expression of STAT6 in macrophages in vitro. **A** Levels of IL-6, TNF, and MCP1 production in macrophages isolated from wild-type mice (females/20 weeks) following in vitro treatment with ruxolitinib were determined utilizing CBA and flow cytometry. **B** The expression of phosphorylated (p-) and unphosphorylated STAT1 and STAT6 in macrophages was assessed by western blotting. **C** Amounts of STAT1 and STAT6 determined by densitometry of the protein bands from two experiments. **β**-Actin was the loading control. The symbols R 0 µM, R 1 µM, R 5 µM and R 10 µM correspond to the various concentrations of ruxolitinib at 0 µM, 1 µM, 5 µM and 10 µM, respectively. The data are representative of 2–3 independent experiments with similar results. *p < 0.05, **p < 0.01, ***p < 0.001, ****p < 0.0001

To investigate how ruxolitinib can affect macrophage functions, macrophages isolated from WT mice were treated with ruxolitinib for 3 days. Thereafter, cytokine levels in the culture supernatant and the related protein expression levels in macrophages were determined. We found that macrophages in the presence of ruxolitinib produced lower levels of the proinflammatory cytokines IL-6 and TNF as well as the chemokine MCP1. The levels of these proinflammatory cytokines decreased concomitantly with increased concentrations of ruxolitinib (Fig. 6A).

We were intrigued by our data on the M1-M2 genotype shift and IRF4 activation in ruxolitinib-treated ARE-Del^+/−^ mice. Previous studies have reported that IRF4 expression requires STAT6 pathways that are well known to drive macrophage M2 polarization [48, 49]. We speculated that STAT6 expression/activation can account for the PC macrophage polarization in ruxolitinib-treated ARE-Del^+/−^ mice. Indeed, our western blotting data demonstrated that pSTAT1 and STAT1 were inhibited in macrophages treated with ruxolitinib, while STAT6 was activated concomitantly, thus facilitating M2 macrophage polarization (Fig. 6B, C). In summary, our data indicated that ruxolitinib can inhibit the production of proinflammatory cytokines and promote the activation of anti-inflammatory signaling in macrophages, thus supporting our model of M2 macrophage polarization and disease reduction.

## Discussion

This study was conducted to determine the therapeutic efficacy of a JAK 1/2 inhibitor in a murine model of PBC. Experimental studies from clinical specimens and from animal models of human PBC have documented a major role of IFNs in the pathogenesis of this disease. For example, Takii et al. reported that the expression levels of type I IFNs were significantly higher in the portal tract and liver parenchyma of patients with PBC than in patients with autoimmune hepatitis and chronic hepatitis C [24]. Our previous study corroborated the key roles of type I and type II IFNs in the pathogenesis of PBC as noted using the ARE-Del murine model of autoimmune cholangitis [26, 38]. The downstream effects of IFNs are driven by the JAK/STAT pathway, resulting in a rapid membrane-to-nucleus signaling cascade. Following expression by immune cells, IFNs bind to their cognate receptors, thus triggering the expression of IFN-responsive target genes that have a critical impact on immune responses and autoimmunity. The distinct physiological outcomes depend upon the corresponding ligands and the context of the microenvironment [27].

Dysregulation of the JAK/STAT pathway has been shown to be involved in many immunological defects, including the enhancement of inflammatory cytokines in autoimmune diseases [13, 50], and has thus become one of the key therapeutic targets for inflammatory and autoimmune diseases. The clinical efficacy of JAK inhibitors has been demonstrated in a number of autoimmune diseases [32–34]. Since a significant role of IFNs in PBC has been well documented in both human and animal models, we hypothesized that JAK1/2 inhibitors can be a promising clinical intervention for patients with PBC. To address this issue, we chose ruxolitinib, an FDA-approved JAK1/2 inhibitor that can block both the type I and type II IFN-induced JAK/STAT pathways, and examined its effects using the established ARE-Del mouse model of PBC. ARE-Del mice exhibit prolonged and chronic overexpression of IFNγ and develop high titers of AMA, portal inflammation, and biliary pathology along with a female predominance, which are attributes that resemble human PBC. Here, we examined the effect of ruxolitinib in ARE-Del^+/-^ mice on liver pathology, AMA levels, immune cell populations and the expression of proinflammatory and anti-inflammatory cytokine genes. We further examined the probable immunological mechanism underlying these salient findings. The data that we obtained allow us to gain further insights into the mechanism of PBC in addition to potentially identifying new therapeutic targets for this disease.

In this study, liver pathology was significantly improved after the administration of ruxolitinib, highlighted by marked reductions in portal inflammation and small bile duct damage (Fig. 1C, D). We further explored the underlying immunological mechanism of the changes in liver pathology. Persistent chronic IFNγ levels impact immune cell populations in ARE-Del mice, including elevated CD4^+^ T-cell-mediated Th1 responses and female-prevalent excessive GC formation with enhanced Tfh and GC responses [26, 38]. These immunological features are documented to be involved in the pathogenesis of autoimmune cholangitis [26, 38]. In this study, we paid special attention to the changes in specific immune cell subpopulations. Splenic CD4^+^ T, GC B and Tfh cells were markedly decreased after ruxolitinib treatment, and the splenic Treg frequency was increased (Figs. 3 and 4). These findings are of paramount significance and are consistent with the results of our previous passive transfer studies. Thus, passive transfer of splenic CD4^+^ T cells from ARE-Del^−/−^ to B6/Rag1^−/−^ mice induces pathological changes that are very similar to those seen in liver tissues from PBC patients [26]. The current data support our previous report that deletion of the *Ifnar1* gene suppressed the accumulation of both Tfh and GC B cells in ARE-Del mice. Together, our data demonstrate that splenic CD4^+^ T, GC B and Tfh cells are critical in the induction of autoimmune cholangitis. We suggest that the decreased liver pathology could be attributed to the suppression of splenic CD4^+^ T, GC B and Tfh cells upon ruxolitinib treatment. Notably, ruxolitinib did not suppress splenic Treg cells but increased their numbers. Since IL-2 is the key cytokine driving Treg cell proliferation, differentiation, and function [51], we conducted an in vitro culture study with splenic CD4^+^ T cells isolated from ARE-Del^+/−^ mice in the presence/absence of ruxolitinib and assayed IL-2 expression. We found that ruxolitinib enhances IL-2 production by splenic CD4^+^ T cells from ARE-Del^+/−^ mice (Fig. 3C). The finding that ruxolitinib attenuates graft-versus-host disease in mice by increasing the frequency of Treg cells [52] supports our finding. Moreover, the lower relative expression of proinflammatory cytokines, including IFNγ and IL-6, in the liver tissues of ruxolitinib-treated mice suggested that cytokine-mediated liver inflammation was inhibited by ruxolitinib (Fig. 2).

AMA is a serological hallmark of PBC that is present in over 95% of patients with PBC [53] and is readily detectable in sera from ARE-Del^+/−^ mice. We compared the serum levels of AMA before and after ruxolitinib treatment. AMA levels were markedly decreased in the treatment group compared to the control group (Fig. 4B), likely due to the decrease in splenic GC B and Tfh cells, which are cell lineages that affect the production of autoantibodies.

Although the adaptive immune system has long been identified as the chief immunological arm in the pathogenesis of human PBC, the innate immune system has also been implicated in the pathogenesis of PBC [53, 54]. There is a higher level of innate immune system activation in PBC patients than controls, as exemplified by the higher levels of cytokines produced by peripheral monocytes from patients with PBC [55]. Monocyte-derived macrophages from patients with PBC generated a proinflammatory cytokine burst in the presence of AMA and apoptotic bodies from small biliary epithelial cells, which indicates that in such a microenvironment, monocyte-derived macrophages can be polarized to M1 [56].

Macrophages are one of the major physiological targets of IFNγ effector action, and IFNγ has been historically identified as the predominant macrophage-activating factor. Hence, we examined the changes in macrophages in the liver and PC. Our data revealed that M1 macrophages were predominant in the liver and PC in ARE-Del^+/−^ mice but not in WT mice. There were more M2 macrophages in WT mice than in ARE-Del^+/−^ mice (Fig. 5A, C). This phenomenon can be accounted for by the capacity of IFNγ to shift the polarization of macrophages toward proinflammatory M1 macrophages. Liver and PC macrophages exhibited a significant decrease in cell numbers and a dramatic shift from an M1 to an M2 phenotype following ruxolitinib therapy (Fig. 5B, D). Our data are supported by a study of ruxolitinib on hemophagocytic lymphohistiocytosis in mice, which showed that liver tissue injury subsided with a decrease in the number of infiltrative inflammatory macrophages and an increase in the number of substituted activated macrophages [57]. Our in vitro study also confirmed that ruxolitinib treatment can lower the levels of IL-6, TNF and MCP1 (Fig. 6A), factors that can contribute to the development of M1 macrophages. In addition, the exploration of a specific signaling pathway indicated that the level of IRF4 expression was markedly increased upon ruxolitinib treatment compared to that in control and WT mice. A series of studies demonstrated that IRF4 was the key downstream transcription factor that controlled M2 gene expression [47, 48], which reinforces our findings. The results from immunoblotting substantiated that pSTAT1 and STAT1 were inhibited while STAT6 was activated in macrophages after administration of ruxolitinib (Fig. 6B, C), thus suggesting that M2 macrophage polarization occurs through the STAT6-IRF4 signaling pathway. Our data were further strengthened by other studies that confirmed that STAT6 drives macrophage M2 polarization [48, 49].

Generally, macrophages are not considered to be the original culprit in the pathogenesis of PBC, but they can mediate tissue damage and disease perpetuation. Macrophages have a dual role, either promoting inflammation or suppressing inflammation, that appears to hinge on distinct cytokines present in the microenvironment, exerting different actions in infectious diseases, neoplastic diseases and autoimmune diseases [58]. Thus, the manipulation of upstream cytokines to divert macrophage function may be a preemptive and promising therapeutic approach in PBC. The contributory role of macrophages in the pathogenesis of PBC is evident [56]. We believe that blocking the production of IFNγ, the upstream proinflammatory cytokine polarizing M1 macrophages, can improve autoimmune cholangitis in ARE-Del^+/−^ mice. From this point of view, this study provides a novel perspective for the treatment of PBC. Further studies are warranted to substantiate the effects of ruxolitinib in other diseases in which macrophages exhibit a pathogenic role. It is clearly of paramount importance to determine whether the findings derived from our mouse model can be translated to the bedside.

In addition to ARE-Del mice, there are other animal models mimicking human PBC phenotypes, such as NOD.c3c4 mice, a dominant-negative form of TGFβ receptor type II (dnTGFβRII) mice, IL-2Rα^−/−^ mice and a xenobiotic-induced PBC model [59]. We are cognizant that there is no perfect animal model that fully recapitulates human PBC. As in many other autoimmune diseases, the complexity of PBC involves genetic predisposition, environmental factors and abnormal immunity. However, we believe that ARE-Del mice are distinctly valuable in this study, as this model exhibits the immunological and liver-specific pathological features of human PBC, including multilineage immune dysregulation, elevated AMA, bile duct destruction, elevated IFNs and a female predominance. We will continue to apply this ARE-Del animal model to reveal the mechanisms underlying chronic IFN**γ** activation in liver injury and inflammation and to define the immune players involved in hepatocyte and biliary damage. The data in this study demonstrated that inhibition of the JAK/STAT pathway by ruxolitinib alleviates biliary pathology via suppression of T cells, B cells and macrophages. It will be of interest to examine whether ruxolitinib treatment also affects other downstream signaling pathways, such as STAT1 activation in hepatocytes and liver inflammation. Further studies on the therapeutic effect of ruxolitinib in other animal models of PBC will broaden the application of JAK/STAT inhibitors in the clinical management of PBC. Future work is needed to determine its translational application in PBC patients.

**Fig. 7:**
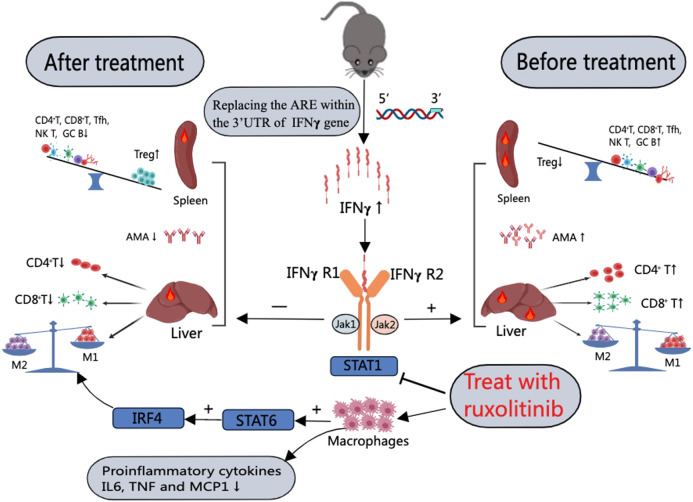
Schematic representation of the possible mechanisms of the effect of JAK inhibitors in ameliorating autoimmune cholangitis in ARE-Del mice. ARE-Del mice generated by replacement of the AU-rich element (ARE) in the 3′ UTR of IFNγ mRNA with random nucleotides exhibit increased chronic expression of IFNγ. The elevation of IFNγ levels activates the JAK-STAT pathway, resulting in (1) an imbalance between splenic Treg cells and CD4^+^ T cells, CD8^+^ T cells, Tfh cells, GC B cells, and NK T cells; and (2) an increase in the number of hepatic CD4^+^ T cells, CD8^+^ T cells and M1 macrophages. Treatment of ARE-Del mice with the JAK inhibitor ruxolitinib resulted in a decrease in splenic CD4^+^ T cells, CD8^+^ T cells, germinal center B cells, and Tfh cells and hepatic CD4^+^ T cells and CD8^+^ T cells but a significant increase in splenic Treg cells. Furthermore, M1 macrophages were polarized to M2 macrophages through activation of the STAT6-IRF4 pathway. In addition, ruxolitinib treatment also reduced the production of the proinflammatory cytokines IL-6 and TNF as well as the chemokine MCP1 in macrophages in vitro. Overall, modulation of the JAK/STAT pathway by a JAK inhibitor alleviated liver pathology and reduced AMA levels in ARE-Del mice. (+ indicates activation; ⎯ and ⊢ indicate inhibition)

In summary, our study demonstrates that effective modulation of the JAK-STAT pathway and inhibition of IFNγ gene expression results in the suppression of splenic CD4^+^ T, CD8^+^ T, GC B, and Tfh cells and hepatic CD4^+^ T cells and CD8^+^ T cells but a significant increase in splenic Treg cells. In addition, the reduction in macrophages in the liver and PC and the polarization of M1 macrophages to M2 macrophages through activation of the STAT6-IRF4 pathway eventually led to alleviation of liver pathology and lowered AMA levels in ARE-Del^+/−^ mice (Table 1 and Fig. 7). Our data provide solid experimental evidence for the efficacy of ruxolitinib in the therapeutic intervention of autoimmune cholangitis. Furthermore, our discovery of the effects of JAK inhibitor modulation of the STAT6-IRF4 pathway on macrophage polarization opens up a novel strategy for the clinical management of PBC and other immunological disorders in which macrophages exhibit a pathogenic role.

**Table 1:** Summary of the immunological changes between ARE-Del^+/−^ mice treated with ruxolitinib and control mice

Characteristic features of ARE-Del^+/−^ mice	Control group	Treatment group
Small bile duct damage	Aggravated	Attenuated
Portal inflammation	Aggravated	Attenuated
AMA	↑	↓
Hepatic CD4+ T	↑	↓
Hepatic CD8+ T	↑	↓
Splenic CD4+ T	↑	↓
Splenic CD8+ T	↑	↓
Splenic Treg	↓	↑
Splenic NK T	↑	↓
Splenic GC B	↑	↓
Splenic Tfh	↑	↓
Liver macrophages	↑/M1	↓/M2
PC macrophages	↑/M1	↓/M2
Relative expression of inflammatory cytokines (IFN**γ**, IL-6, and TNF**α**) in liver tissues	↑	↓

## Supplementary information

Table S1

Table S2

Table S3

Table S4

Table S5
