# Supplementary material for: Treatment with a JAK1/2 inhibitor ameliorates murine autoimmune cholangitis induced by IFN overexpression
Source: Cell Mol Immunol. 2022 Aug 30;19(10):1130–40. doi: 10.1038/s41423-022-00904-y (PMC9508183; doi:10.1038/s41423-022-00904-y)
Supplement: Supplementary file 1 — Table S1 [file 41423_2022_904_MOESM1_ESM.docx]

**Table S1.** **Liver Histological Scoring Criteria**

| Portal Inflammation | | Lobular inflammation | | Bile duct damage | | Granuloma |
| --- | --- | --- | --- | --- | --- | --- |
| Severity | Frequency | Severity | Frequency | Severity | Frequency | Frequency |
| 0: no change | 0: none | 0: no change | 0: none | 0: no change | 0: none | 0: none |
| 1: minimal | 1: 1%-10% | 1: minimal | 1: 1%-10% | 1: epithelial damage (cytoplasmic change) | 1: 1%-10% | 1: a few |
| 2: mild | 2: 11-20% | 2: mild | 2: 11-20% | 2: epithelial damage  with nuclear change | 2: 11-20% | 2: portal or lobular |
| 3: moderate | 3: 21-50% | 3: moderate | 3: 21-50% | 3: CNSDC | 3: 21-50% | 3: portal and lobular |
| 4: severe | 4: more than 50% | 4: severe | 4: more than 50% | 4: Bile duct loss | 4: more than 50% | 4: marked |
