# Supplementary material for: Treatment with a JAK1/2 inhibitor ameliorates murine autoimmune cholangitis induced by IFN overexpression
Source: Cell Mol Immunol. 2022 Aug 30;19(10):1130–40. doi: 10.1038/s41423-022-00904-y (PMC9508183; doi:10.1038/s41423-022-00904-y)
Supplement: Supplementary file 2 — Table S2 [file 41423_2022_904_MOESM2_ESM.docx]

**Table S2.** **Antibodies for Flow Cytometry**

| Fluorochrome | Antigens | Company | Catalog number | Clone number |
| --- | --- | --- | --- | --- |
| FITC | CXCR5 | BioLegend | 145520 | L138D7 |
| PE | PD-1 | BioLegend | 135206 | 29F.1A12 |
| PerCP | CD4 | BioLegend | 100538 | RM4-5 |
| APC | TCR𝛃 | BioLegend | 109212 | H57-597 |
| PE | GL-7 | BioLegend | 144607 | GL7 |
| PerCP | B220 | BioLegend | 103234 | RA3-6B2 |
| FITC | CD19 | BioLegend | 115506 | 6D5 |
| PerCP | CD8a | BioLegend | 100732 | 53-6.7 |
| APC-Cy7 | CD4 | BioLegend | 100414 | GK1.5 |
| PerCP | F4/80 | BioLegend | 123126 | BM8 |
| PE | CD80 | BioLegend | 104708 | 16-10A1 |
| APC | CD206 | BioLegend | 141708 | C068C2 |
| APC/Fire ^TM^ 750 | TCR𝛃 | BioLegend | 109246 | H57-597 |
| PE-Cy5 | CD19 | BioLegend | 115510 | 6D5 |
| Brilliant Violet 711 ^TM^ | NK1.1 | BioLegend | 108745 | PK136 |
| Brilliant Violet 650 ^TM^ | CD8a | BioLegend | 100742 | 53-6.7 |
| Brilliant Violet 570 ^TM^ | CD4 | BioLegend | 100542 | RM4-5 |
| Alexa Fluor 647 | IFN𝛄 | BioLegend | 505814 | XMG1.2 |
| PE | IL17A | BioLegend | 506904 | TC11-18H10.1 |
| FITC | CD95 | BD Biosciences | 554257 | Jo2 |
| PE-Cy^TM^ 7 | TCR𝛃 | BD Biosciences | 560729 | H57-597 |
| APC-Cy7 | CD19 | BD Biosciences | 557655 | 1D3 |
| PE | NK1.1 | BD Biosciences | 553165 | PK136 |
| Alexa Fluor 488 | IRF4 | Cell Signaling | 33859S | E8H3S |
| Alexa Fluor 488 | FOXP3 | Invitrogen | 53-5773-82 | FJK-16s |
