# Supplementary material for: Treatment with a JAK1/2 inhibitor ameliorates murine autoimmune cholangitis induced by IFN overexpression
Source: Cell Mol Immunol. 2022 Aug 30;19(10):1130–40. doi: 10.1038/s41423-022-00904-y (PMC9508183; doi:10.1038/s41423-022-00904-y)
Supplement: Supplementary file 3 — Table S3 [file 41423_2022_904_MOESM3_ESM.docx]

**Table S3.** **Primers for Real-time PCR**

| Genes | | Primers (5’-3’) |
| --- | --- | --- |
| GAPDH | Forward | CATGGCCTTCCGTGTTCCTA |
|  | Reverse | CCTGCTTCACCACCTTCTTGAT |
| IFN𝛄 | Forward | TAGCCAAGACTGTGATTGCGG |
|  | Reverse | AGACATCTCCTCCCATCAGCAG |
| IL6 | Forward | TCCATCCAGTTGCCTTCTTG |
|  | Reverse | TTCCACGATTTCCCAGAGAAC |
| TNFα | Forward | AAGCCTGTAGCCCACGTCGTA |
|  | Reverse | AGGTACAACCCATCGGCTGG |
| TGFβ | Forward | AACAATTCCTGGCGTTACCTT |
|  | Reverse | CTGCCGTACAACTCCAGTGA |
