# Supplementary material for: Treatment with a JAK1/2 inhibitor ameliorates murine autoimmune cholangitis induced by IFN overexpression
Source: Cell Mol Immunol. 2022 Aug 30;19(10):1130–40. doi: 10.1038/s41423-022-00904-y (PMC9508183; doi:10.1038/s41423-022-00904-y)
Supplement: Supplementary file 4 — Table S4 [file 41423_2022_904_MOESM4_ESM.docx]

**Table S4.** **Antibodies for Western Blotting**

| Antibody | Company | Catalog number |
| --- | --- | --- |
| Stat1 Antibody | Cell Signaling | 9172S |
| Phospho-Stat1 (Ser727) Antibody | Cell Signaling | 9177S |
| Stat6 Antibody | Cell Signaling | 9362S |
| Phospho-Stat6 (Tyr641) (D8S9Y) Rabbit mAb | Cell Signaling | 56554S |
| β-Actin (D6A8) Rabbit mAb (HRP Conjugate) | Cell Signaling | 12620S |
| Anti-rabbit IgG, HRP-linked Antibody | Cell Signaling | 7074S |
