# Supplementary material for: Treatment with a JAK1/2 inhibitor ameliorates murine autoimmune cholangitis induced by IFN overexpression
Source: Cell Mol Immunol. 2022 Aug 30;19(10):1130–40. doi: 10.1038/s41423-022-00904-y (PMC9508183; doi:10.1038/s41423-022-00904-y)
Supplement: Supplementary file 5 — Table S5 [file 41423_2022_904_MOESM5_ESM.docx]

**Table S5. Total splenic mononuclear cell numbers between treatment and control group**

| Animal | Treatment group (×10^6^) | Control group (×10^6^) |
| --- | --- | --- |
| 1 | 59 | 76 |
| 2 | 71 | 89 |
| 3 | 31 | 55 |
| 4 | 40 | 134 |
| 5 | 43 | 51 |
| 6 | 71 | 67 |
| 7 | 55 | 52 |
| 8 | 41 | 71 |
| 9 | 27 | 69 |
| 10 | 27 | 51 |
| 11 | 21 |  |
